# Acute inflammation is associated with lower muscle strength, muscle mass and functional dependency in male hospitalised older patients

**DOI:** 10.1371/journal.pone.0215097

**Published:** 2019-04-15

**Authors:** Jessamine Y. J. Liu, Esmee M. Reijnierse, Jeanine M. van Ancum, Sjors Verlaan, Carel G. M. Meskers, Andrea B. Maier

**Affiliations:** 1 Department of Medicine and Aged Care, The Royal Melbourne Hospital, The University of Melbourne, Melbourne, Victoria, Australia; 2 Department of Human Movement Sciences, Vrije Universiteit, Amsterdam Movement Sciences, Amsterdam, The Netherlands; 3 Department of Internal Medicine, Section of Gerontology and Geriatrics, Amsterdam UMC, Vrije Universiteit Amsterdam, Amsterdam, The Netherlands; 4 Department of Rehabilitation Medicine, Amsterdam UMC, Vrije Universiteit Amsterdam, Amsterdam, The Netherlands; Universita degli Studi di Napoli Federico II, ITALY

## Abstract

**Background:**

Hospitalisation is associated with adverse health outcomes including loss of muscle strength, muscle mass and functional decline, which might be further aggravated by acute inflammation. This study aimed to determine whether acute inflammation, as denoted by C-reactive protein (CRP), is associated with muscle strength, muscle mass and functional dependency in hospitalised older patients.

**Methods:**

The observational, prospective EMPOWER study included 378 hospitalised patients aged 70 years and older. As part of the hospital assessment, 191 patients (50.5%) had CRP measured. Muscle strength and mass were measured using handheld dynamometry and bioelectrical impedance analysis respectively. Activities of Daily Living (ADL) were assessed using Katz score and Instrumental ADL (IADL) by Lawton and Brody score. Linear regression analyses and logistic regression analyses were performed stratified by sex and adjusted for age and comorbidities.

**Results:**

Mean age was 79.7 years (SD 6.4) and 50.8% were males. On admission and discharge, males with elevated CRP had significantly lower handgrip strength and lower absolute muscle mass compared with males with normal CRP and those with no CRP measured. At three months post-discharge, males with elevated CRP were more likely to be ADL dependent than those with normal CRP and with no CRP measured. In females, no associations were found between CRP and muscle strength, muscle mass, ADL or IADL.

**Conclusions:**

Hospitalised older male patients with acute inflammation had lower muscle strength at admission and discharge and lower absolute muscle mass at admission and higher ADL dependency at three months post-discharge.

## Introduction

Hospital admissions of older adults have more than doubled in the last decade [1]. Hospitalisation is associated with adverse outcomes such as delirium, falls, loss of muscle strength and muscle mass, and reduced functional ability to perform activities of daily living (ADL) [2–6]. Older patients have less physiological reserve compared to younger individuals, so even a small reduction in muscle strength and functional ability may have a significant influence on their functional independence and living situations [7,8].

Inflammation has been shown to be an independent risk factor for disability and mortality in hospitalised older patients [9,10] assuming a catabolic effect on skeletal muscle metabolism [9,11]. In community-dwelling older adults, low-grade chronic systemic inflammation has been associated with low muscle strength and low physical function in both cross-sectional and longitudinal studies [9,11–17]. In the hospitalised adult population, patients with severe sepsis accompanied by high-grade inflammation were found to have significantly lower muscle strength and exercise capacity at hospital discharge and performed less physical activity at three months post-discharge compared to healthy control subjects [18]. In inpatients with non-critical illness and mild inflammation, significantly lower handgrip strength was found compared to those without inflammation [19]. The association between acute inflammation and muscle mass has not been studied, nor has the association between acute inflammation and muscle strength and ADLs in hospitalised older patients.

This study aimed to determine the association of C-reactive protein (CRP) during hospitalisation, as an indicator of acute inflammation, with 1) muscle strength, muscle mass, ADL and Instrumental ADL (IADL) dependency at admission, 2) with muscle strength and muscle mass on discharge and 3) with changes in ADL and IADL dependency three months after discharge in hospitalised older patients.

## Materials and methods

### Study design

The Evaluation of Muscle Parameters in a Prospective cohort of Older patients at clinical Wards Exploring Relations with bed rest and malnutrition (EMPOWER) study is an observational, prospective inception cohort study. Between April 2015 and December 2015, patients aged 70 years and older who were admitted to the acute admission, internal medicine, trauma and orthopaedic wards at the VU University Medical Center, Amsterdam, The Netherlands, were considered eligible to participate. Patients were excluded if unwilling or unable to give informed consent, had an expected length of stay less than 24 hours, were unable to be assessed within 48 hours after admission, required isolation room, or were terminally ill. In total, 838 patients were screened and 378 patients were included in the study [20]. Baseline assessment was performed within 48 hours of admission including measurement of muscle strength, muscle mass, ADL and IADL scores. The second assessment of muscle strength and muscle mass was performed at seven days after the initial assessment or on the day of discharge if the length of stay was less than seven days. A follow-up telephone survey was conducted to assess ADL and IADL scores three months post discharge. The study design was reviewed and approved by the medical ethics committee of the VU University Medical Center, Amsterdam, The Netherlands. Informed consent was obtained from all included patients.

### Patient characteristics

Medical records were used to collect patient demographics and clinical profile including age, sex, comorbidities, length of stay and type of admission (acute versus elective). The number of comorbidities was defined as the number of any diseases they were suffering from at hospital admission. The major categories of comorbidities were presented i.e. coronary heart disease, chronic obstructive pulmonary disease, stroke, malignancy, diabetes and heart failure. Other characteristics were obtained during an interview with the patient including living situation (independent/dependent), current smoking (yes/no), current use of alcohol (yes/no) and risk of malnutrition measured by the Short Nutritional Assessment Questionnaire, SNAQ [21] (low risk of malnutrition SNAQ score 0–1 points and high risk of malnutrition SNAQ score ≥2 points) [22]. Weight in kilograms (kg) was measured using a weighing chair. When weight was unable to be measured, self-reported weight was used. Height in centimetres (cm) was estimated using knee height and calculated using the Longitudinal Aging Study Amsterdam (LASA) formula (male = 74.48 + (2.03 * knee height)–(0.15 * age), female = 68.74 + (2.07 * knee height)–(0.16 * age)). Body mass index (BMI) was calculated as weight divided by height squared (kg/m^2^).

### C—reactive protein

CRP levels were measured using immunoturbidimetric assay on Roche/Hitachi cobas c 501 analysers (Roche Diagnostic Corp., Indianapolis, USA). The highest CRP values during hospitalisation were recorded. CRP was categorized into three groups: elevated CRP ≥10 mg/L, normal CRP <10mg/L [23] and no CRP measured. Of those with elevated CRP, the severity of inflammation was defined as mild ≥10 and <50 mg/L, moderate ≥50 and <100 mg/L, and severe >100 mg/L [19]. Of the 378 patients, CRP was measured in 191 patients as part of the hospital assessment based on the clinical indication of the physician to suspect inflammation.

### Muscle strength

Muscle strength was determined using handgrip strength (HGS) and was measured using a hydraulic hand dynamometer (Jamar, Sammons Preston, Inc. Bolingbrook, IL, USA). Patients were asked to sit in an upright position with their elbow flexed to 90 degrees and unsupported. If patients were unable to sit in an upright position, HGS was measured while patients were reclined at 30 degrees in bed. Patients were encouraged to squeeze maximally. Two attempts on each hand side were made and the maximal value of all four measurements was used for analysis and expressed in kilograms.

### Muscle mass

Muscle mass was measured using direct-segmental multi-frequency bioelectrical impedance analysis (DSM-BIA) [24] (InBody S10, Biospace Co., Ltd, Seoul, Korea). Patients were asked to lie down in a supine position, spread their arms to about 15 degrees angle, arms not touching the trunk and spread legs to shoulder width, thighs not touching each other. Patients were asked not to move during the measurements. If patients were not able to lie down completely flat, measurements were performed in a maximum supine position that could be reached. BIA measurements were not performed when the patient had a pacemaker or other implanted electronic device (n = 25, 6.6%) and when electrodes could not be placed on fingers or ankles (n = 21, 5.6%). Muscle mass measures included absolute muscle mass (skeletal muscle mass (SMM) in kilograms) and relative muscle mass (SMM as a percentage of the body weight, %).

### Activities of daily living and instrumental activities of daily living

Katz index [25] was used to assess ADL dependency with the score ranging from 0 to 6 points; a higher score implying higher independence. ADL score was dichotomized into independent (score of 6 points) and dependent (score of 5 points or less) [26]. Lawton and Brody scale [27] was used to assess IADL dependency with the score ranging from 0 to 8 points; a higher score implying higher independence. IADL score was dichotomized into independent (score of 8 points) and dependent (score of 7 points or less) [26,28]. Change in ADL and IADL was defined as the three-month post discharge score minus the admission score.

### Statistical analysis

Numerical data with a normal distribution were expressed as mean with standard deviation and as median with interquartile range if not normally distributed. Categorical variables were presented as numbers and percentage.

The association between CRP and muscle strength and muscle mass was analysed using linear regression analysis using categorical dummy variables for CRP (elevated vs. normal vs. no CRP measured) and CRP severity (normal vs. mild vs. moderate vs. severe) and was adjusted for age and comorbidities. For visualisation purposes using GraphPad Prism version 7.0c for Mac OS X (GraphPad Software, La Jolla California USA), an Analysis of Variance (ANOVA) was performed to obtain estimated means and standard error for the association between CRP and muscle strength and muscle mass, adjusted for age and comorbidities.

The association between CRP (elevated vs. normal vs. no CRP measured) and ADL and IADL dependency was analysed using logistic regression analysis with adjustments for age and comorbidities (model 1), and model 1 plus baseline ADL or IADL dependency (dependent versus independent) (model 2). Linear regression analysis was performed to assess the association between CRP and changes in ADL and IADL scores between admission and three months post discharge, adjusting for age and comorbidities (model 1), and model 1 plus baseline ADL or IADL score (model 2).

All analyses were stratified by sex as sex was found to be a significant effect modifier in the associations between CRP and muscle measures, ADL and IADL dependency. All data were analysed using Statistical Package for the Social Science (SPSS) version 24 (IBM Corp, Armonk, New York). P values <0.05 were considered statistically significant.

## Results

**Fig 1:**
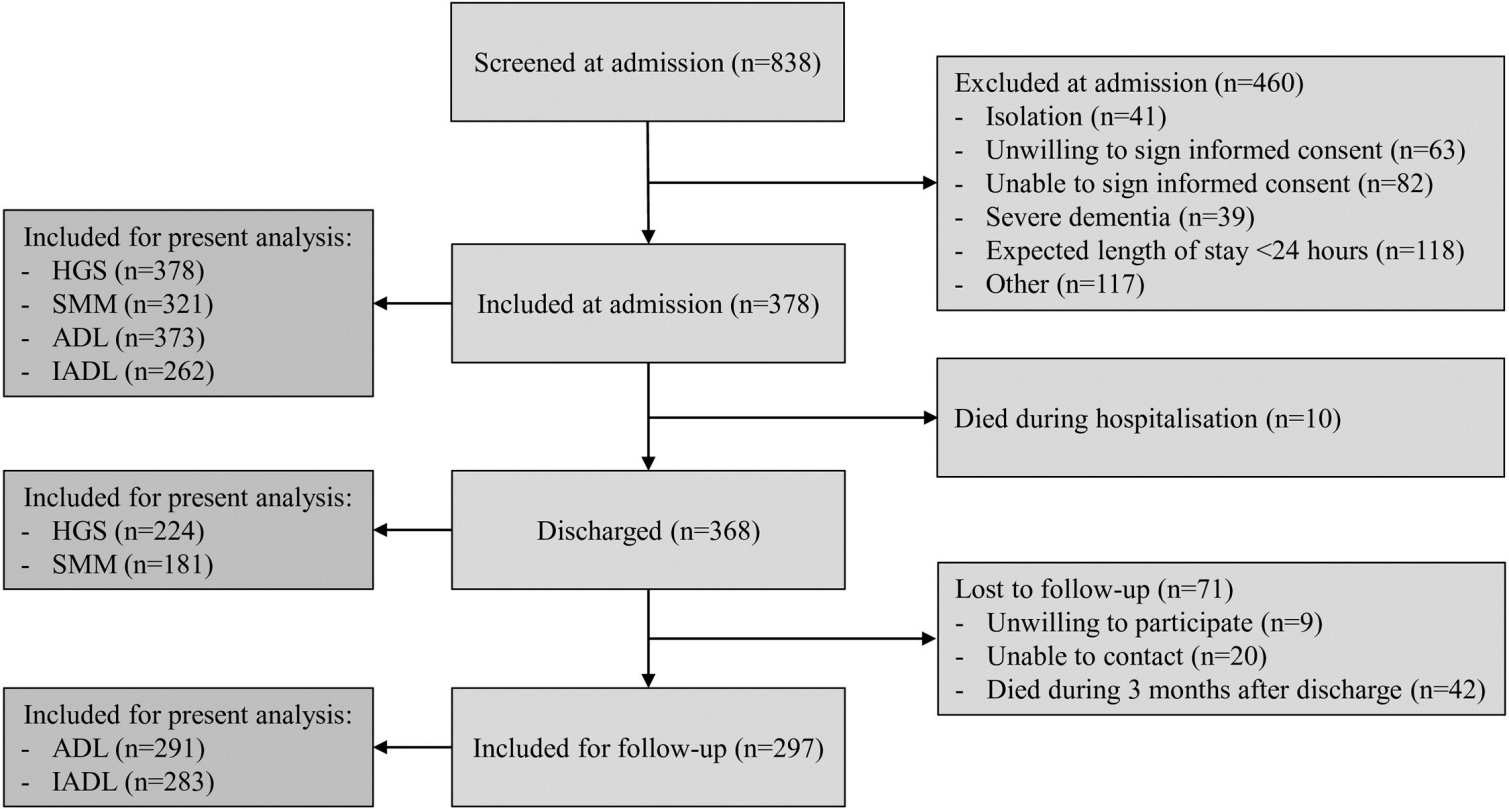
Flowchart of patient screening, inclusion, follow-up of the EMPOWER cohort and patients included for the present analyses (adapted from Verlaan et al. 2017 [20]).

Fig 1 shows the flowchart of patient screening, inclusion, follow-up, and patients included for the present analyses. Table 1 shows the baseline characteristics of patients stratified by sex. The mean age was 79.7 years (SD 6.4) and 49.2% were females. The majority of patients were living independently prior to hospitalisation (94.4%) and were acutely admitted (84.7%). The median length of stay was 5 days (IQR 3–8 days). Of the 191 patients with measured CRP, 155 had elevated CRP (81%) and 36 (19%) had normal CRP.

**Table 1:** Baseline characteristics of inpatients included in the EMPOWER cohort.

	**N**	**Total** **(n = 378)**	**N**	**Males (n = 192)**	**N**	**Females** **(n = 186)**
**Characteristics**						
Age, years	378	79.7 ± 6.4	192	79.1 ± 6.3	186	80.3 ± 6.5
Living independently, n (%)	376	355 (94.4)	192	185 (96.4)	184	170 (92.4)
Weight, kg	378	73.1 ±17.1	192	77.4 ± 14.6	186	68.7 ± 18.3
Height, cm	378	168.5 ± 9.5	192	175.2 ± 6.9	186	161.5 ± 6.3
BMI, kg/m^2^	378	25.8 ± 5.7	192	25.2 ± 4.4	186	26.4 ± 6.8
Risk of malnutrition, n (%)	377	130 (34.5)	192	70 (36.5)	185	60 (32.4)
Comorbidities, median (IQR)	376	3 (2–5)	192	3 (2–5)	184	3 (2–5)
Coronary heart disease, n (%)	377	46 (12.2)	192	27 (14.1)	185	19 (10.3)
COPD, n (%)	377	47 (12.5)	192	27 (14.1)	185	20 (10.8)
	**N**	**Total** **(n = 378)**	**N**	**Males (n = 192)**	**N**	**Females** **(n = 186)**
Stroke, n (%)	377	64 (17.0)	192	38 (19.8)	185	26 (14.1)
Malignancy, n (%)	377	134 (35.5)	192	75 (39.1)	185	59 (31.9)
Diabetes, n (%)	377	83 (22.0)	192	52 (27.1)	185	31 (16.8)
Heart failure, n (%)	377	61 (16.2)	192	36 (18.8)	185	25 (13.5)
Length of stay, median (IQR)	378	5 (3–8)	192	5 (3–7)	186	5 (3–9)
Acute admission, n (%)	378	320 (84.7)	192	167 (87.0)	186	153 (82.3)
**Inflammation**						
Elevated CRP^a^, n (%)	191	155 (81.2)	95	77 (81.1)	96	78 (81.3)
**Muscle measures**						
Handgrip strength, kg	378	20.6 ± 9.8	192	26.1 ± 9.9	186	14.9 ± 5.6
SMM, kg	321	26.1± 6.0	158	29.8 ± 5.6	163	22.5 ± 3.8
SMM, %	321	36.4 ± 6.0	158	39.1 ± 5.0	163	33.7 ± 5.7
**Functional dependency**						
ADL score, median (IQR)	373	5 (3–6)	192	6 (3–6)	181	5 (3–6)
ADL dependent, n (%)	373	188 (50.4)	192	91 (47.4)	181	97 (53.6)
IADL score, median (IQR)	262	7 (4–8)	131	6 (4–7)	131	7 (5–8)
IADL dependent, n (%)	262	176 (67.2)	131	99 (75.6)	131	77 (58.8)
**Functional dependency 3 months post discharge**						
ADL score, median (IQR)	291	5 (4–6)	140	6 (4–6)	151	5 (4–6)
ADL dependent, n (%)	291	151 (51.9)	140	64 (45.7)	151	87 (57.6)
IADL score, median (IQR)	283	6 (3–7)	135	6 (4–7)	148	6 (3–8)
IADL dependent, n (%)	283	217 (76.7)	135	107 (79.3)	148	110 (74.3)

All variables are presented as mean (SD), unless otherwise indicated. N: number of patients. BMI: Body Mass Index. CRP: C-reactive protein. SMM: Skeletal muscle mass. ADL: Activities of Daily Living. IADL: Instrumental Activities of Daily Living.

^a^Elevated CRP is defined as CRP ≥10 mg/L.

COPD, Chronic obstructive pulmonary disease.

### CRP and muscle measures

#### Admission

**Fig 2:**
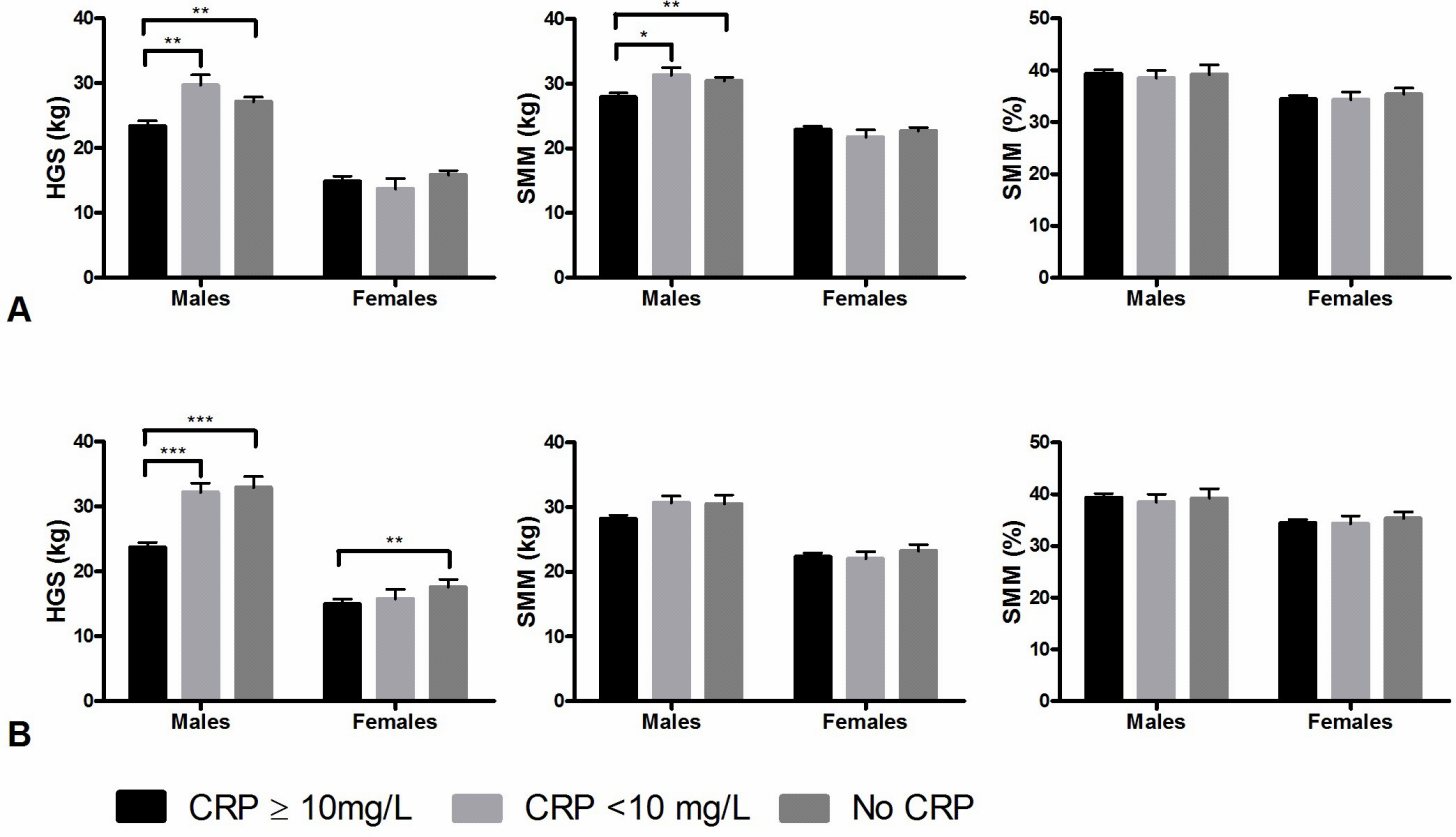
Association between CRP groups and muscle strength and muscle mass. Muscle measures are represented as standardised means with standard errors, adjusted for age and comorbidities. A: admission. B: discharge. HGS: handgrip Strength. SMM: Skeletal Muscle Mass. Level of significance: * p<0.05, ** p<0.01, *** p<0.001.

Fig 2A shows the association between CRP and muscle measures at admission. Males with elevated CRP had significantly lower HGS (23.4 kg, SE 0.8) compared with males with normal CRP (29.7 kg, SE 1.6) and those with no CRP measured (27.1 kg SE 0.7). Males with elevated CRP had significantly lower absolute SMM (27.9 kg SE 0.6) compared with those with normal CRP (31.3 kg SE 1.2) and those with no CRP measured (30.5 kg SE 0.5). CRP was not associated with relative SMM. In females, no associations were found between CRP and HGS and muscle mass.

#### Discharge

Fig 2B shows that males with elevated CRP had significantly lower HGS (23.7 kg, SE 0.7) compared to those with normal CRP (32.2 kg, SE 1.5) and those with no CRP measured (32.9 kg, SE 1.7). Females with elevated CRP had lower HGS (15.0 kg, SE 0.7) compared to those with normal CRP (15.7 kg, SE 1.4) and no CRP measured (17.7 kg, SE 1.2). In both males and females, no associations were found between CRP and absolute and relative SMM at discharge.

### CRP level and muscle measures

**Fig 3:**
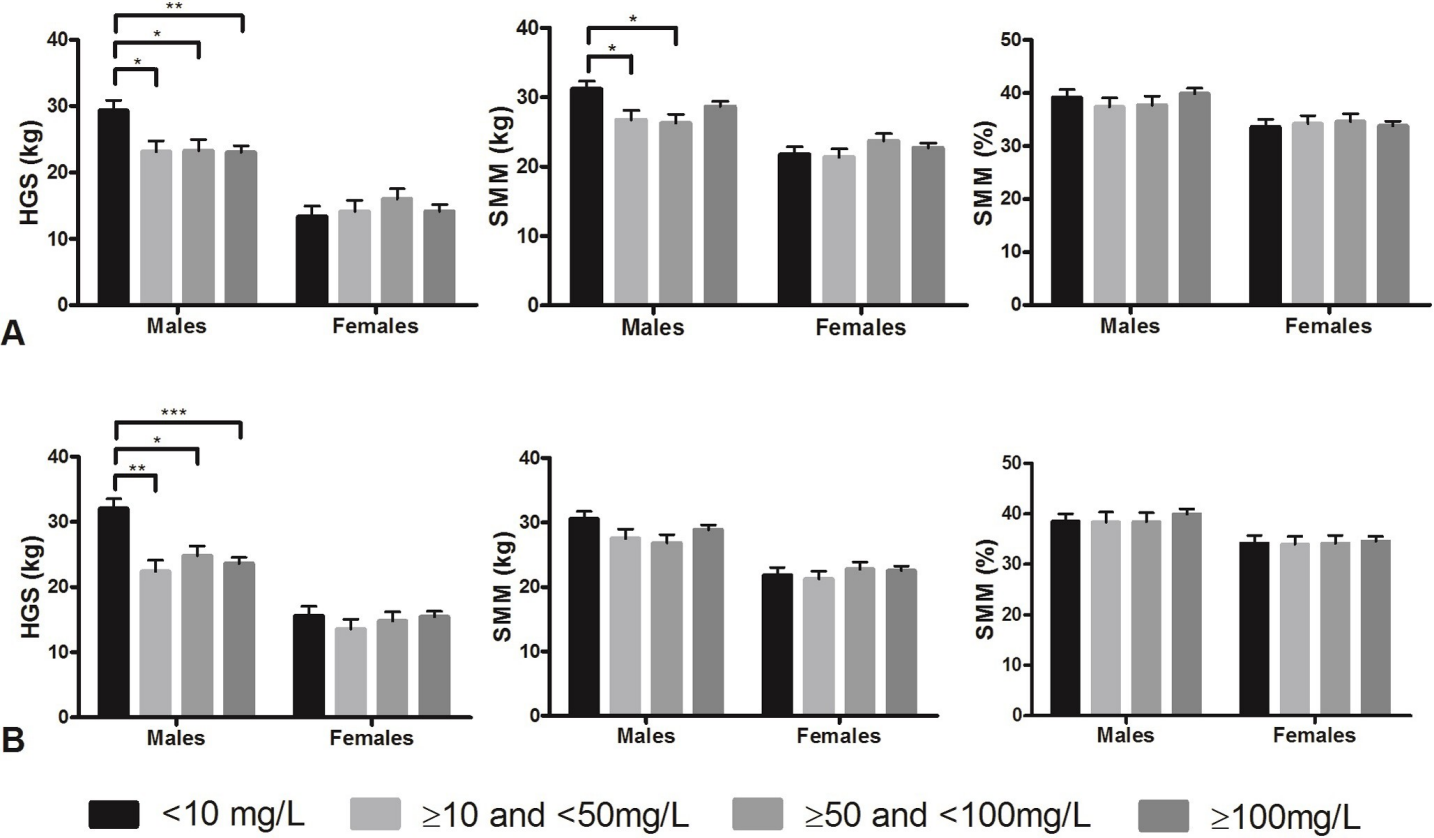
Association between severity of CRP and muscle strength and muscle mass. Muscle measures are represented as standardised means with standard errors, adjusted for age and comorbidities. A: admission. B: discharge. HGS: handgrip Strength. SMM: Skeletal Muscle Mass. CRP severity: <10mg/L (normal CRP), ≥10mg/L and <50mg/L (mild inflammation), ≥50mg/L and <100mg/L (moderate inflammation), ≥100mg/L (severe inflammation). Level of significance: * p<0.05, ** p<0.01, *** p<0.001.

Fig 3 shows the association between the severity of inflammation, expressed by CRP level and muscle strength and muscle mass. In male patients, mild, moderate, and severe inflammation, were associated with significantly lower handgrip strength compared to those with normal CRP on admission and at discharge. Mild and moderate inflammation groups were associated with significantly lower absolute SMM compared to those with normal CRP on admission. There was no significant difference between mild, moderate and severe inflammation in the association with handgrip strength and SMM in males and females.

### CRP and functional dependency

Table 2 shows the association between CRP and ADL and IADL. At admission, males with elevated CRP had non-significantly higher ADL dependency compared to those with normal CRP and no CRP measured. Males with elevated CRP were more likely to be dependent in their ADLs at 3 months post-discharge than those with normal CRP and with no CRP measured, this was not statistically significant after adjusting for baseline ADL status. In males, CRP was not associated with IADL dependency on admission or at three months post-discharge and no association of CRP with the change in ADL or IADL was observed.

**Table 2:** Association between CRP and activities of daily living and instrumental activities of daily living.

	ADL dependency						IADL dependency					
	Admission		3 months post-discharge		Change in score		Admission		3 months post-discharge		Change in score	
	OR (95% CI)	p	OR (95% CI)	p	Beta (SE)	p	OR (95% CI)	p	OR (95% CI)	p	Beta (SE)	p
**Males–Elevated CRP vs. normal CRP**												
Model 1	3.36 (0.96–11.7)	0.057	6.82 (1.32–35.3)	**0.022**	0.40 (0.53)	0.448	1.28 (0.30–5.50)	0.744	2.21 (0.46–10.6)	0.321	-0.90 (0.63)	0.157
Model 2	N/A		4.51 (0.81–25.2)	0.086	-0.42 (0.42)	0.315	N/A		1.98 (0.26–15.3)	0.511	-1.13 (0.58)	0.053
**Males–Elevated CRP vs. no CRP measured**												
Model 1	1.46 (0.76–2.80)	0.261	2.23 (1.01–4.89)	**0.046**	0.12 (0.32)	0.707	1.30 (0.53–3.19)	0.571	2.08 (0.73–5.97)	0.171	-0.31 (0.39)	0.428
Model 2	N/A		1.79 (0.77–4.20)	0.180	-0.30 (0.25)	0.239	N/A		3.19 (0.74–13.8)	0.121	-0.71 (0.37)	0.057
**Females–Elevated CRP vs. normal CRP**												
Model 1	1.05 (0.34–3.24)	0.939	1.47 (0.46–4.75)	0.516	0.30 (0.47)	0.527	1.99 (0.51–7.72)	0.323	0.78 (0.19–3.16)	0.730	1.03 (0.48)	**0.037**
Model 2	N/A		1.93 (0.51–7.30)	0.332	0.19 (0.39)	0.627	N/A		0.36 (0.06–2.09)	0.254	0.88 (0.54)	0.105
**Females–Elevated CRP vs. no CRP measured**												
Model 1	1.55 (0.81–2.97)	0.185	1.33 (0.64–2.79)	0.446	0.06 (0.31)	0.860	1.44 (0.61–3.41)	0.408	1.53 (0.65–3.62)	0.334	0.48 (0.39)	0.218
Model 2	N/A		1.18 (0.49–2.85)	0.720	-0.10 (0.25)	0.703	N/A		0.68 (0.19–2.50)	0.565	0.37 (0.38)	0.333

CRP: C-reactive protein. ADL: Activities of Daily Living. IADL: Instrumental Activities of Daily Living. OR: odds ratio. 95% CI: 95% confidence interval. N/A: not applicable. Model 1: adjusted for age and comorbidities. Model 2: model 1 + baseline ADL/IADL.

In females, no association was observed between CRP and ADL or IADL on admission and at three months post-discharge. Females with elevated CRP had an increase in IADL score indicating increased independence compared to those with normal CRP at three months post-discharge, this finding was not present when comparing females with elevated CRP to those with no CRP measured.

## Discussion

In male hospitalised patients 70 years and older, acute inflammation was associated with lower muscle strength and lower absolute muscle mass on admission, and with significantly lower muscle strength at discharge, independent of the severity of inflammation. Acute inflammation was associated with higher ADL dependency on admission and at three months after discharge in males. In females, acute inflammation was only associated with lower muscle strength on discharge and increase in IADL independence score at three months post-discharge.

### CRP and muscle measures

While chronic inflammation and its association with muscle strength and muscle mass has been studied extensively in community-dwelling older adults [9,11–15,17], the impact of acute inflammation on muscle measures and functional dependency in hospitalised older adults has not been studied. In the present study, hospitalised older male patients with acute inflammation had on average 5 kilograms lower muscle strength compared to male patients without acute inflammation. This effect size is higher than previously found in a younger hospitalised adult population [19], where inflammation was associated with lower handgrip strength of 1–3 kg [19]. Older patients were shown to have a more pronounced systemic response to pathophysiological stress than younger patients [29] and this could lead to more impact on muscle measures. The difference in muscle strength between older and younger patients could also be due to lower physiological reserve and therefore less compensatory mechanisms in older patients [29,30] causing more damage to the muscles with the same degree of inflammation. On the counterpart, it has also been postulated that an inflammatory environment is needed at the site of muscle damage to stimulate muscle repair [31].

The association between inflammation and muscle strength and muscle mass was independent of the severity of inflammation. This was consistent with a previous study result that even mild inflammation was associated with lower handgrip strength [19]. Also in situ and in vitro studies suggest that muscle characteristics might be independent on the level of inflammation [32,33] It is possible that acute inflammation leads to a cascade of events [34] that mediates the change in muscle strength and muscle mass, and this cascade of events was independent of the severity of inflammation, and this will require further exploration.

The mechanism by which CRP is associated with lower handgrip strength and muscle mass could be two-fold. Firstly, inflammation is known to have a catabolic effect on muscle [9,11]. In addition, inflammation may be a reflection of more severe and systemic illness affecting muscle measures.

Clinical characteristics of the EMPOWER cohort were similar to the GLISTEN cohort of acutely hospitalized older patients in regards to age, sex and BMI [35]. However, the EMPOWER cohort had a shorter length of stay and a higher proportion of acute admissions compared to the GLISTEN study. The prevalence of sarcopenia was 34.7% at hospital admission, similar to the EMPOWER cohort [36], and the incidence of sarcopenia was 14.7% in the GLISTEN cohort.

### CRP and ADL/IADL

Acute inflammation was associated with higher ADL dependency in male patients; this association was present on admission and at three months post-discharge. Our results were in line with a previous cross-sectional population study [37], where chronic inflammation was associated with functional dependency in ADL and IADL. Functional impairments influence patients’ level of care needs and residential options [7,8,38]. This could have implications in terms of targeted post-acute hospitalisation rehabilitation, to optimise patient functional independence and reduce the admission rate to residential aged care facilities.

### Sex differences

The significant association between CRP and muscle measures and functional dependency was found only in males but not in females. This sex difference in the association between inflammation and muscle measures and functional dependency had not been reported previously. One possible explanation is that in response to acute inflammation, the endocrine mediators including sex hormones may produce different biological response in males and females, where there is reduced systemic inflammatory response in females than in males [29,39,40].

### Strength and limitations

To the best of our knowledge, this is the first study to report the association between acute inflammation and muscle strength muscle mass and ADL/IADL in a hospitalised older patient population. In this study, CRP was used as the biomarker of acute inflammation. CRP was collected as part of routine clinical care and hence where not indicated, CRP was not evaluated in such individual patients. Patients with no CRP measured during their hospitalisation showed a similar pattern of muscle characteristics as patients with normal CRP which confirmed proper identification practice of patients eligible for CRP assessments. Biomarkers such as butyryl-cholinesterase, but also cytokines such as tumour necrosis factor alpha and interleukin-6 have been examined as chronic inflammatory biomarkers in sarcopenia [9,11,15,16,41]. Our database was limited to biomarkers measured in routine clinical care. Measurement of handgrip strength may be subject to effort dependent variation. Encouragement was standardised to minimise this potential limitation. BIA measurement could be influenced by the hydration status of patients: overestimation of muscle mass could have occurred in fluid overload or underestimation in dehydration [42].

## Conclusions

Hospitalised older male patients with acute inflammation had significantly lower muscle strength on admission and discharge, significantly lower absolute muscle mass on admission, and significantly increased activities of daily living dependency at three months post-discharge. In females, acute inflammation was associated with lower muscle strength on discharge. This has implications for targeted rehabilitation follow up in patients with acute inflammation, to optimise their muscle strength, muscle mass and restore the premorbid level of function. Longitudinal studies would be required to assess the trajectory of muscle strength and muscle mass following acute inflammation in the short and medium term following discharge.

## Supporting information

S1 Appendix STROBE checklist for cohort studies. (PDF)
